# The Convergence of FTIR and EVs: Emergence Strategy for Non-Invasive Cancer Markers Discovery

**DOI:** 10.3390/diagnostics13010022

**Published:** 2022-12-21

**Authors:** Le-Wei Wong, Siow-Hui Mak, Bey-Hing Goh, Wai-Leng Lee

**Affiliations:** 1 Department of Medicine, University of Malaya Medical Centre, Kuala Lumpur 59100, Malaysia; 2 School of Science, Monash University Malaysia, Subang Jaya 47500, Malaysia; 3 Biofunctional Molecule Exploratory (BMEX) Research Group, School of Pharmacy, Monash University Malaysia, Subang Jaya 47500, Malaysia; 4 College of Pharmaceutical Sciences, Zhejiang University, Hangzhou 310027, China

**Keywords:** extracellular vesicles, infrared spectroscopy, FTIR, biomarker, cancer detection, machine learning, chemometrics, automated diagnosis

## Abstract

In conjunction with imaging analysis, pathology-based assessments of biopsied tissue are the gold standard for diagnosing solid tumors. However, the disadvantages of tissue biopsies, such as being invasive, time-consuming, and labor-intensive, have urged the development of an alternate method, liquid biopsy, that involves sampling and clinical assessment of various bodily fluids for cancer diagnosis. Meanwhile, extracellular vesicles (EVs) are circulating biomarkers that carry molecular profiles of their cell or tissue origins and have emerged as one of the most promising biomarkers for cancer. Owing to the biological information that can be obtained through EVs’ membrane surface markers and their cargo loaded with biomolecules such as nucleic acids, proteins, and lipids, EVs have become useful in cancer diagnosis and therapeutic applications. Fourier-transform infrared spectroscopy (FTIR) allows rapid, non-destructive, label-free molecular profiling of EVs with minimal sample preparation. Since the heterogeneity of EV subpopulations may result in complicated FTIR spectra that are highly diverse, computational-assisted FTIR spectroscopy is employed in many studies to provide fingerprint spectra of malignant and non-malignant samples, allowing classification with high accuracy, specificity, and sensitivity. In view of this, FTIR-EV approach carries a great potential in cancer detection. The progression of FTIR-based biomarker identification in EV research, the rationale of the integration of a computationally assisted approach, along with the challenges of clinical translation are the focus of this review.

## 1. Introduction

Surgical biopsy and imaging analysis, together with histopathological analyses, are deemed as the gold standard diagnostic techniques for solid tumors in clinical settings. A comprehensive histological assessment of biopsied tissues enables the characterization of pathologies [1]. However, the procedure is known to be invasive, time-consuming, and labor-intensive; patients often experience postoperative pain and discomfort and are often associated with high infection risk. Moreover, due to tumoral heterogeneity, a single tissue biopsy may not be sufficient to provide a representative profile of the tumor; however, the destructive nature of biopsy testing makes it impractical to perform multiple tissue biopsies or routinely obtain tissue biopsies from different sites for a comprehensive tumor characterization, including histological or morphological, immunohistochemical, and molecular profiling, or to obtain information of the real-time status of the disease [2]. Histological interpretation is extremely subjective and operator-dependent, which might result in ambiguous conclusions owing to the experience required, subjectivity of interpretation, and categorization skills [3]. These factors impose limits on the accuracy of cancer diagnosis, particularly in the early stages of pathology. These reasons have fueled the research trend towards the discovery and development of minimally invasive cancer diagnostic techniques, such as liquid biopsy. Liquid biopsy, which is currently employed as an alternative diagnostic approach to tissue biopsy, includes the collection and clinical assessment of various bodily fluids, such as blood, urine, saliva, and semen. These biofluid samples can provide real-time information on numerous circulating biomarkers, such as circulating tumor cells (CTCs), circulating cell-free DNA (cfDNA), microRNA, tumor-educated platelets, and different extracellular vesicle (EV) subpopulations [2,4]. 

Early cancer detection can potentially increase patient survival and quality of life. Therefore, identifying cost-effective biomarkers with pronounced sensitivity and specificity is essential for cancer diagnosis and monitoring to facilitate the appropriate therapy. The progression of fundamental molecular understanding and advancement of molecular techniques in the characterization of extracellular vesicles (EVs) have opened a new avenue of potentially utilizing EVs as biomarkers for diagnosing and monitoring diseases, including cancer [5]. By leveraging the very much unique characteristics of EVs—heterogeneous populations of cell-secreted membranous nanoparticles that carry molecular footprints of their cell or tissue of origin [6], the abundance of circulating EVs in biofluids, and the distinctive molecular profiles of their cell of origin—a wealth of biological information can be obtained from their membrane surface markers and cargo loaded with biologically active molecules, such as nucleic acids, proteins, lipids, etc., making them exploitable for clinical applications, especially clinical cancer diagnostic and therapeutic purposes [7]. Given their essential roles in facilitating cell-cell signaling and communication, EVs are also involved in pathological processes by delivering oncogenic cargo to recipient cells—both healthy and cancer cells—and elicit phenotypic alterations or neoplastic transformations that promote cancer tumorigenesis, angiogenesis, and metastasis [8]. Nevertheless, using multiplex assays, most EV-based cancer studies have focused on investigating a single cancer marker, such as protein, RNA, miRNA, mutant DNA allele, or other molecular signatures [9]. Tumors with high genetic or phenotypic heterogeneity may not be able to obtain a complete molecular profile using these techniques. As none of the candidate biomarkers have been validated as satisfactory for current clinical applications such as diagnosis and prognosis, EVs are still rarely applied in clinical settings. Thus, there remains a gap of knowledge in fine tuning the approach to achieve rather precise and accurate characterization outcomes [10]. 

Fourier-transform infrared spectroscopy (FTIR) has been employed to study cancer-derived EVs [11]. Exploiting the very nature of its principle of detection—non-destructive and label-free molecular profiling of the nucleic acid, lipid, and protein content that requires only minimal sample preparation needed for analysis—FTIR is set to emerge as a diagnostic tool for rapid compositional characterization of EVs. In addition, FTIR spectroscopy is sensitive to the conformational changes in biomolecules, such as alterations in the secondary structure of EV-associated protein, which is valuable information in clinical practice [10]. In the analysis of EVs, FTIR spectroscopic analysis relies on the interaction between mid-infrared (IR) radiation and molecular bonds, and the biochemical profile of a particular EV sample is then reflected by the resultant absorption spectrum. Nonetheless, the heterogeneity of EV subpopulations might result in FTIR spectra that are highly diverse and complex, making the interpretation challenging and complicated. In order to resolve this issue, computational-assisted FTIR spectroscopy has been implemented in many studies to produce fingerprint spectra of malignant and non-malignant samples, enabling classification with high accuracy, specificity, and sensitivity.

The purpose of this review is to explore FTIR spectroscopy’s applications in EV analysis for cancer diagnosis. We provide a brief overview of different types of EVs and their diagnostic values in cancer. Next, we discuss some theoretical considerations of IR radiation and how FTIR spectroscopy could be used to discover EV biomarkers. We also provide examples of computational-aided IR spectra analysis, including multivariate approaches and machine learning algorithms. Lastly, we conclude the current obstacles and potential strategies before this diagnostic tool can be implemented to analyze EV biomarkers for cancer screening in clinical practices.

## 2. Survey Methodology

### 2.1. Eligibility Criteria

In this article, we review recent studies involving spectroscopic analysis of EVs and cancer detection. The search was limited to articles published in English from 2012 onward. Articles were manually filtered after evaluating their title, abstract, and discussion to confirm their relevance to this review. Additional publications were also obtained from the reference lists of the selected articles. We excluded conference abstracts, case reports, commentaries, editorials, unpublished manuscripts, and letters to the editor. Collectively, it is hoped that a review of the current role of IR spectroscopy in the analysis of cancer-derived EVs can promote the discovery of cancer markers and the development of a non-invasive method for cancer detection.

### 2.2. Database and Search Strategies

A Boolean search was performed on Google Scholar, PubMed, and ScienceDirect. Combinations of various search terms like (“spectroscop*” OR “IR spectroscop*” OR “infrared spectroscop*”) AND (“EV*” OR “extracellular vesicle*” OR “microvesicle*” OR “exosome*”) AND (“cancer*” OR “malignan*” OR “tumo*r” OR “onco*”) were used. In addition, manual searches had also been conducted.

## 3. Extracellular Vesicles (EVs)

### 3.1. Subtypes and Biogenesis of EVs

**Figure 1:**
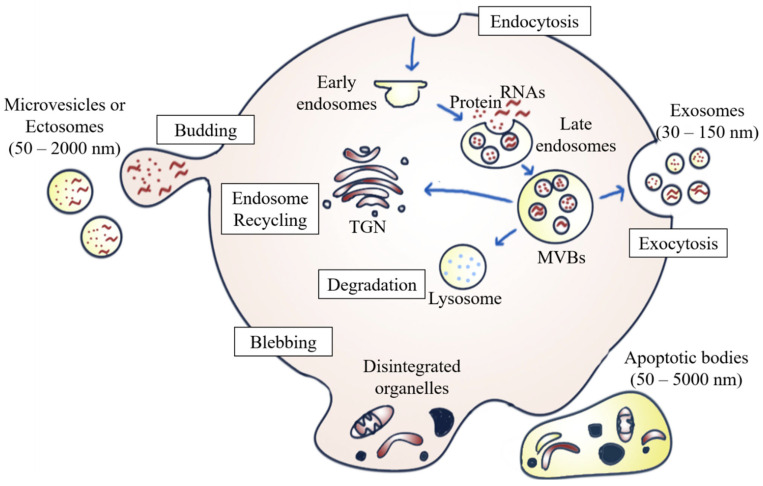
Biogenesis of different EV subpopulations. Exosomes originate from the fusion of the inward budding of MVBs formed via the endosomal pathway in the plasma membrane. ILVs are either secreted to the extracellular environment as exosomes upon fusion of multivesicular bodies (MVBs) with the plasma membrane, degraded by lysosomes, or recycled by trans-Golgi network (TGN). Ectosomes are formed by outward budding from the plasma membrane, whereas apoptotic bodies are generated by membrane blebbing and fragmentation of apoptotic cells.

EVs are heterogeneous populations of cell-secreted spherical bilayer nanoparticles that are mainly involved in intercellular communication [6]. Exosomes, ectosomes, and apoptotic bodies are the common EV subpopulations. They are different from one another in terms of size, biogenesis, function, and cargo content [7]. In brief, exosomes (30–150 nm) are small EVs arising from the endosomal network [7,12]; ectosomes, (50–2000 nm), which are also known as microvesicles (MVs), originate from the plasma membrane’s outward budding and dividing; whereas apoptotic bodies (50–5000 nm) are secreted by dying cells in the apoptosis process [12] (Figure 1).

Exosome formation occurs when exosomal vesicles form through the inward budding and invagination of the plasma membrane, which then mature into vesicles containing multivesicular bodies (MVBs). Once the MVBs fuse with the plasma membrane, the intraluminal vesicles (ILVs) inside the MVBs are then secreted into the extracellular milieu as exosomes. This mechanism is referred to as exosome biogenesis and sets MVBs apart from other forms of EVs released by the outward budding of the plasma membrane [13]. The MVBs can be either degraded by lysosomes, recycled via the trans-Golgi network, or fused with the cell surface to release exosomes via exocytosis [14]. The biogenesis of exosomes involving the endosomal sorting complex required for transport complexes (ESCRTs), together with the accessory proteins such as vacuolar protein sorting-associated protein 4 (VPS4) and ALG-2 interacting protein X (ALIX), participate in the protein sorting and formation of ILVs [6]. In addition to the ESCRT-dependent pathway, exosomes can also be formed through an ESCRT-independent biogenesis pathway involving ceramides [15].

In addition to internal budding, EVs can also bud outward directly from the plasma membrane, forming ectosomes. Similar to exosomes, the ESCRT pathway is also involved [16]. The development of ectosomes results from constantly changing interactions between phospholipid translocation and contraction of cytoskeletal structures. Phosphatidylserine translocation to the membrane’s outer leaflet can trigger membrane budding and vesicle formation. The budding process then ends with the contraction of cytoskeletal structures caused by actin-myosin interactions [12].

There are multiple steps in the programmed cell death process. Apoptosis usually begins with nuclear chromatin condensation, irregular bulging of the plasma membrane, and finally, the breakdown of cellular content into discrete apoptotic bodies. For example, S1PR1, S1PR3, CD63, LAMP1, and stress-associated proteins such as HSP70 are more highly expressed by apoptosomes during apoptosis than by normal exosomes, indicating that the biogenesis of apoptotic bodies is associated with sphingosine-1-phosphate—sphingosine-1-phosphate receptors (S1P–S1PRs) complex signaling, which can further induce inflammatory mediators in macrophages. Apoptosomes can enter phagocytes by endocytosis or by fusing with the plasma membrane of macrophages, thus enhancing the mRNA transcription of inflammatory cytokines, such as interleukin (IL)-1β. Therefore, it can be concluded that apoptotic bodies are involved in the pathogenesis of inflammatory diseases, including cancer [17].

### 3.2. Roles of EVs in Cancers

EVs are well known to facilitate intercellular communication by transferring a variety of complex molecular cargo, including protein, lipid, nucleic acid, and metabolites, and consequently exert a wide range of biological and functional effects in EV-recipient cells upon their uptake [18]. In brief, EVs can interact with recipient cells via ligand-receptor signaling at the cell surface or fuse with plasma membranes, followed by EV uptake via receptor-mediated endocytosis or macropinocytosis [19]. Intercellular communication has been found to play an essential role in tumorigenesis, which allows cancer cells to create a favorable immune microenvironment for cancer progression by interacting with their surroundings [18]. Accumulating evidence has proven that tumor-derived EVs are involved in the modulation of cancer hallmarks, including induction of replicative cell immortality, tumor invasion, metastasis, angiogenesis, immune evasion, as well as drug resistance.

Cancer cells were reported to induce the expression of telomerase in recipient normal fibroblasts by transferring human telomerase reverse transcriptase (hTERT) mRNA through exosome cargo, hence enabling the cells to acquire the cancer stem cell phenotype and the capability of replicative immortality [20]. In addition, tumor-derived EVs are essential mediators in creating a tumor-supportive stroma. For instance, normal fibroblasts have been shown to differentiate into cancer-associated fibroblasts (CAFs) in response to acquiring oncogenic cargo from EVs, especially oncogenic miRNAs [21,22]. Activated CAFs, in turn, regulate and promote the proliferation and invasion of cancer cells by secreting significant amounts of biological factors, such as growth factors, pro-inflammatory cytokines, and chemokines (e.g., IL-6 and transforming growth factor-beta, TGF-β), to the tumor microenvironment [23], as well as by transmitting EV cargo [24,25].

Angiogenesis, which involves the formation of new blood vessels from existing vasculature, can facilitate the delivery of materials, such as nutrients and oxygen, and is especially vital in supporting the growth of cancer cells [26]. Tumor-derived EVs have also been shown to induce angiogenic switch from physiologic to pathologic angiogenesis through the significant release of pro-angiogenic factors, such as matrix metalloproteinases (MMPs), vascular endothelial growth factor (VEGF), and non-coding RNAs (ncRNAs, e.g., microRNAs and long non-coding RNAs), to activate pro-angiogenic activities in the adjacent vasculature, leading to pathologic neovascularization that supports tumor growth [27]. Furthermore, tumor-derived EVs are well known for evasion from immune surveillance [28]. Kim et al. [29] reported that exosomes originating from non-small cell lung cancer expressed functional PD-L1, which inhibited T-cell activity beyond the local microenvironment. In addition, tumor-derived EVs are capable of promoting immunosuppression by transferring immunosuppressive cytokines and ncRNAs [28].

Drug resistance is a significant clinical obstacle to cancer treatment. EVs released by drug-resistant cells are believed to play a critical role in the maintenance and transmission of drug resistance in various cancers, including pancreatic, liver, glioblastoma, etc. [30]. Cancer cells can acquire multidrug resistance by directly exporting or sequestering cytotoxic drugs via EVs, and also confer a resistance phenotype to drug-sensitive cancer cells by transmitting cargo such as mRNAs, ncRNAs, lipids, and drug-resistance-related proteins (e.g., anti-apoptotic proteins, drug efflux transporters, etc.) that interfere with cell cycle control and induce anti-apoptotic signaling in recipient sensitive cells [30].

### 3.3. Diagnostic Values of EVs in Cancer

EVs exist in numerous biological fluids, including blood [31,32], saliva [4,33], urine [34], bronchoalveolar lavage fluid (BALF) [35], semen [36], etc. The lipid bilayer membrane of EVs protects the enclosed cargo of protein, lipid, nucleic acid, etc., from external enzymatic degradation, hence ensuring the integrity and functionality of the cargo throughout the entire cargo delivery process [37]. Their inherent stability and capability to retain the integrity of their contents enables researchers to access the molecular contents of their parental cells [38]. Therefore, researchers can obtain vital molecular information about disease statuses by targeting the EV-derived cargo, which reflects the intracellular status and physio-pathological state of the parental tumor cells. This information enables comprehensive tumor genome and transcriptome characterization, as well as the detection of rapid changes in the tumor microenvironment due to their short half-lives, ranging from a couple of minutes to a few hours before being taken up by recipient cells [39]. In contrast to tissue biopsy, EVs are suitable for sequential collection and obtaining tumor status in real time because of their minimally invasive sampling procedure and easy accessibility. EV-associated proteins and RNAs have received the most attention among the components of EVs as tumor biomarkers for diagnosing and monitoring cancer development [40].

#### 3.3.1. EV-Associated Proteins

Many studies have proven that the protein composition differs significantly between EVs in malignant and non-malignant samples, as well as between different types of malignant samples, which allows EV protein to be a potential biomarker for clinical diagnostic purposes. EVs are capable of carrying tumor-specific proteins. Various EV-associated proteins have been reported to be significantly upregulated in cancer patients compared to healthy controls. Odaka et al. [41] demonstrated elevated levels of platelet marker (CD41 and CD61)-positive and tetraspanin (CD63)-positive EVs in sera isolated from pancreatic ductal adenocarcinoma (PDAC) patients, particularly sera CD63^+^-EVs that showed promising diagnostic performance in separating PDAC patients from healthy subjects, with an area under the curve (AUC) value of 0.846. These findings were in line with those of Huang et al. [42], who reported elevated expression levels of plasma-derived exosomes and the chaperone protein mortalin in cancer patients, which promoted proliferation and induced epithelial-mesenchymal transition (EMT) of cancer cells.

Differential expression of EV proteins in different types of malignant cells have been investigated in several studies, thereby increasing the potential of EVs as an attainable source of cancer biomarkers. The cell-specific protein profiles of oral squamous cell carcinoma (OSCC), PDAC, and melanoma brain metastasis cell lines have been revealed via proteomic profiling. The identified proteins were found to be involved in pathological processes such as angiogenesis, cell proliferation, migration, etc. Remarkably, despite the common proteins shared by the examined cell lines, the EVs showed individual profiles that were characteristic to each cell line. In short, OSCC-derived EVs were enriched in EGFR and ITGB4; PDAC-derived EVs showed upregulated expression of mucins, including MUC5AC, MUC5B, and MUC16; while LAMA1 and LAMB1 were highly expressed in EVs isolated from melanoma brain metastasis cells [43].

The discrimination of cancer subtypes based on variations in the protein composition of EVs may prove to be clinically useful. Rontogianni et al. [44] investigated the use of EV-associated proteins (proteins, phosphoproteins, and protein kinases) for breast cancer detection and molecular subtyping. Their findings highlighted that the proteomic and phosphoproteomic profiles of EVs derived from different cancer cell lines were obviously distinct, hence reflecting the unique characteristics of each breast cancer subtype. Briefly, triple negative breast cancer (TNBC), which is greatly invasive and metastatic, released EVs that were abundant in proteins associated with angiogenesis (PLAU, ADAM9, EPHA2), cell migration (VIM, AXL), and integrin-binding (ITGA5, TIMP2). On the contrary, HER2-positive breast cancer, which is proliferative in nature, secreted EVs with the hallmark proteins involved in ERBB signaling (GRB7, SHC1), translation (EIFs), and axon guidance (DNM2, PIK3R1). 

Meanwhile, Tian et al. distinguished metastatic breast cancer patients from non-metastatic breast cancer patients and healthy controls based on the expression levels of eight protein markers on plasma-derived EVs (CA 15-3, CA 125, CEA, HER2, EGFR, PSMA, EpCAM, and VEGF) using machine learning assisted-thermophoretic aptasensor (TAS) [45]. Due to the poor sensitivity and specificity of single protein markers to discriminate between different patient groups, the authors established an EV diagnostic signature for metastatic breast cancer based on the combination of the eight protein markers. Linear discriminant analysis (LDA) showed that the diagnostic signature permitted robust classification, with considerably high accuracy to discriminate breast cancer patients from the healthy controls (area under the precision-recall curves, AUPRC = 0.9912), and metastatic from non-metastatic patients (AUPRC = 0.9433).

#### 3.3.2. EV-Associated RNAs

There is significant evidence showing the capability of using RNA profiles to reveal the signature of different cell types, indicating that EV-associated RNAs (exRNAs) may play a role in diagnostic evaluation, especially in precancerous or cancer early diagnosis. This is because exRNAs encapsulated within the bilayer lipid structure of EVs are protected from degradation by ribonuclease (RNase), which allows them to be stably detected in biofluids such as plasma or serum. ExRNAs are thus potential candidates as biomarkers for cancer [46]. 

MiRNAs are small non-coding RNAs that regulate gene expression. Therefore, pathology is often associated with dysregulation of miRNA expression through mechanisms such as translocation, amplification or deletion of miRNA genes; up- or downregulation of miRNAs; abnormal epigenetic modifications (e.g., DNA methylation and histone modifications) of miRNA; and impairment of miRNA biogenesis [47]. MiRNA exchange via EVs has thus been reported to play a key role in cancer progression. For example, breast cancer cells could activate normal fibroblasts to CAFs within the tumor microenvironment by transferring miR-125b via EVs to normal fibroblasts [48]. Additionally, Ueta et al. [49] found that the combination of miR-1246 with the biomarkers CEA and CA19-9 in serum EVs showed high diagnostic power in gallbladder cancer. These findings demonstrated the usefulness of EV-associated miRNAs as useful biomarkers for cancer diagnosis. In addition to miRNAs, other smaller RNA families, such as piwi-interacting RNA (piRNA) [50], Y-RNA [51], transfer RNA (tRNA) [52], vault RNA (vRNA) [53] and small nucleolar RNA (snoRNA) [54], have also been discussed in EV-related cancer studies.

Other than small-sized RNA families, larger RNA, such as mRNA, long non-coding RNA (lncRNA), and circular RNA (circRNA), have also been highlighted. It has been suggested that plasma-derived extracellular vesicle long RNA (exLR) could be used as predictive biomarkers for the detection of breast cancer. Su et al. [55] successfully classified breast cancer, benign, and healthy controls based on variations in their exLR profiles with a support vector machine (SVM) model. In the study, the selected exLR markers permitted classification with significantly high accuracy in the training and validation sets, with AUC values of 0.960 and 0.900, respectively; additionally, early stage of breast cancer was identified with AUC value of 0.94. Interestingly, combining the exLR signature with breast cancer imaging improved the differential diagnostic performance of early-stage breast cancer. Yu et al. [56] also established a diagnostic signature comprising eight exLRs characteristic for PDAC based on the profiling of plasma-derived exLRs. Their findings demonstrated outstanding performance in the training, internal validation and external validation cohorts of the SVM based-diagnostic model, with AUC values of 0.960, 0.950 and 0.936, respectively. Additionally, the established diagnostic signature permitted the identification of early stage (I/II) PDAC with an AUC value of 0.949 in the combined three cohorts. Remarkably, the diagnostic signature outperformed the CA 19-9 tumor marker in differentiating PDAC (AUC = 0.931) from controls (AUC = 0.873). The combination of different RNA species, for instance, two mRNAs (KRTAP5-4 and MAGEA3) and a lncRNA (BCAR4) derived from sera EVs, demonstrated excellent predictability of colorectal adenoma, with AUC values of 0.936 and 0.877 in the training and test cohorts, respectively [57]. Furthermore, Lin et al. [58] established that the unique circular RNA (circRNA) expression profiles of breast cancer patients and healthy controls facilitated predictive classification using machine learning models. The chosen circRNAs, which were closely linked to cancer progression, showed promising performance in classification with an AUC value of 0.83.

## 4. Theoretical Considerations of Infrared (IR) Radiation in Biological Studies

### 4.1. Infrared (IR) Spectroscopy

IR spectroscopy measures the interaction between IR radiation and matter. IR radiation is electromagnetic radiation with wavelengths and wavenumbers ranges of 0.78–1000 µm and 12,500–10 cm^−1^, respectively. The IR region in the electromagnetic spectrum can be subdivided into three spectral regions based on the wavelength and wavenumber: near-IR (NIR), mid-IR (MIR), and far-IR (FIR). Table 1 shows the subdivision of IR regions of the electromagnetic spectrum into NIR, MIR, and FIR with their corresponding wavelengths and wavenumbers [59]. Applications of these IR spectral regions in the biological field are briefly discussed in the following sections.

#### 4.1.1. Far-Infrared (FIR)

The FIR region is commonly defined as the region from 400 to 10 cm^−1^. On the other hand, the terahertz (THz) region refers to the spectrum range from 333 to 3.33 cm^−1^, which overlaps with the FIR region. THz and FIR spectroscopy principles are alike; the difference in names results from the instruments used according to experimental requirements [60].

FIR or THz radiation has been widely used in biomedical research, for example, by using cancer cell lines or cancer patients to establish cancer phototherapies [61,62,63]. FIR or THz radiation is highly sensitive to water content, hence making FIR or THz spectroscopy a potential screening tool for cancer diagnosis, based on the difference in the water content and water binding status in normal and cancerous tissues [64]. With increased water content in cancerous tissues compared to that in normal tissues, Vafapour et al. [65] were able to differentiate between healthy and cancerous colon tissues based on variations in their reflectance spectra. Kawashima et al. [66] suggested that the development of fibrous tissue around the malignant liver tumor tissue leads to water loss, thus giving rise to a higher permeability of THz transmission in the imaging experiments. FIR or THz radiation has been proven to be useful in bioimaging. The radiation also exerts beneficial biological effects by producing thermal and non-thermal effects, including strengthening cardiovascular function by enhancing vessel endothelial function, improving vasodilation, a vessel circulation, and angiogenesis; promoting wound healing [67,68]; treating postoperative lymphedema [62,69,70,71]; reducing postoperative pain [72]; suppressing skin photoaging [73,74]; etc.

In addition, FIR is more susceptible to the vibration modes of peptide skeletons and hydrogen bonds compared to MIR spectroscopy, it is therefore considered ideal for examining highly ordered protein structures, such as fibril, gels, and virus-like particles, as well as protein dynamics [75,76]. Several features of peptides and polyamides were linked to distinct modes of vibration according to their characterization based on spectroscopic data. However, proteins such as hemoglobin, lysozymes, and serum albumin showed only weak and broad absorption bands in the FIR region, hence requiring complex and time-consuming calculations and simulations to interpret the data obtained [76].

#### 4.1.2. Mid-Infrared (MIR)

MIR (4000–400 cm^−1^) is commonly used in studying biological samples because most molecule bands, such as proteins, lipids, sugars, and nucleic acids, are present in the MIR region. According to Beer–Lambert’s law, molecular absorbance in this region is proportional to the concentration without light scattering; the measurement of these biological samples can evaluate any change in composition or structure [77]. In the biomedical area, IR spectroscopy, specifically MIR, has been used to investigate large numbers of cells, tissues, and organs, providing qualitative and quantitative information that could be used for detection and classification. This technology can be used in numerous disciplines of biodiagnostics, not only to characterize diseases and monitor drug delivery but also to reveal the biomolecular framework underlying the particular alterations processes and structures. IR spectroscopy could also be applied in forensic science to screen typical human bodily fluids collected from crime scenes as routine confirmation [78]. Notably, FTIR spectroscopy is considered an excellent method to study and analyze biological samples using MIR radiation [79].

#### 4.1.3. Near-Infrared (NIR)

NIR spectroscopy (12,500–4000 cm^−1^) was exclusively employed as an accessory to other optical equipment before the advent of light-fiber optics and monochromator detectors; since then, NIR spectroscopy has become a tool that could be applied in various scientific disciplines, including medicine [80]. NIR applications in bioscience include medical monitoring [81], cell-related studies, analysis of bodily fluids and tissues [82], etc.

NIR calibration models for analyzing the cancer markers of prostate carcinoma (PCa) were among the first applications of NIR spectroscopy in cancer diagnosis [83]. Calibration was performed using a novel adaptive method to attenuate metabolically induced covariance between specific biomolecules in PCa cells. In addition to common bodily fluids such as serum and saliva, NIR can also analyze blood oxygen levels. In studies, higher total hemoglobin and water absorption were determined by analyzing NIR absorption between normal and malignant tissues, followed by weaker signals associated with oxygen saturation in lipids and tissue hemoglobin [84]. Furthermore, NIR spectroscopy has been extensively applied to investigate blood glucose content. A study conducted by Henn et al. found that both NIR and MIR can analyze urea and glucose in hemodialysis monitoring. However, MIR is the better tool for monitoring hemodialysis because MIR, coupled to a multi-reflection attenuated total reflection (ATR) cell, can provide access to more analyses of interest, such as lactate, phosphate, and creatinine [85]. 

With NIR imaging techniques, aqueous solution dispersion can be probed more easily, and changes in structure or concentration in water and proteins can also be monitored simultaneously [60]. However, compared to IR spectroscopy, the strongly overlapping bands generated by NIR spectroscopy are around 10 to 100 times weaker than the corresponding MIR bands [81], resulting in broad absorption profiles that complicate the identification of contributing vibrations [82] and band assignment [81]. Therefore, NIR spectroscopy still faces intense competition from IR spectroscopy, especially in bioanalytical research and the field of medical diagnosis [82].

### 4.2. Biomolecular Vibrations and MIR Spectrum

The absorption bands displayed on an IR spectrum can be assigned to specific molecular vibrations of the sample constituents in which most of the biomolecules absorb energies from MIR [86]. In this IR spectral region, the range of 1800–900 cm^−1^ is called the bio-fingerprint region, where the molecular vibrations in this region are unique for different types of biological samples, including different types of tumors [87,88]. For instance, IR absorption bands of amide I and II observed at 1650 and 1550 cm^−1^, methylene groups of lipids at 1470–1400 cm^−1^, stretching vibrations of phosphodiester groups at 1225 and 1080 cm^−1^, C-OH and C-O stretching of amino acids and carbohydrates at 1155 cm^−1^ and glycogen at 1030 cm^−1^ were reported to discriminate the plasma samples of healthy individuals from those with intraepithelial lesion or malignancy (NILM) and squamous intraepithelial lesion (SIL) [88]. In general, MIR regions including the high wavenumber region, which generally corresponds to stretching vibrations such as C-H, N-H, and O-H, together with the low wavenumber region, which correlate to bending and carbon skeleton fingerprint vibrations, characterize the structure of biological samples [89,90]. Table 2 shows the assignments of the main peaks observed in biological IR spectra, based on the literature.

Many studies have introduced FTIR as a promising biophysical tool for protein structural characterization and monitoring dynamic conformational changes [91]. The IR spectra of protein molecules exhibit many characteristic vibrational frequencies. These vibrational frequencies originate mainly from in-plane C-C stretch, C-N stretch, N-C stretch, N-H stretch and bend, CO stretch and bend, CNC and CCN deformation, and out-of-plane CO and N-H bend and CN torsion [95].

Amino acid residues are connected in proteins or polypeptides via amide bonds. In fact, protein secondary structures are attributed to the hydrogen bonds formed between atoms of the polypeptide backbone [96]. The differential hydrogen bonding in amino acids, together with geometric orientations of amide bonds, give rise to individual secondary structural folding in polypeptides (α-helix, β-sheet, and unordered structures), thereby contributing to resolvable absorption bands in the amide I band corresponding to secondary conformations in polypeptides [97,98,99]. Table 3 shows the assignment of protein secondary structures based on the analysis of the IR amide I band [98,100].

The amide I and II bands are the most conformationally sensitive among the protein bands. The amide I band, which is mainly composed of peptide carbonyl stretching vibration, has been predominantly used as the most sensitive spectral region to access protein conformational information and is less likely to be influenced by the nature of side chains [99]. In contrast, other amide bands are rarely used due to their complexity and are affected by details such as the force field, side chains, and hydrogen bonding [91]. However, the overlapping peaks corresponding to distinct secondary conformations of proteins make band assignment challenging. Thus, the amide I band has to be resolved into multiple individual band components by performing mathematical approaches, including second derivatives and band curve fitting or Fourier self-deconvolution, which correspond to the α-helix, β-sheet, turn, random, etc. of the protein compositions [97,98]. Mathematical and statistical approaches such as multivariate analysis have been increasingly employed in interpreting and revealing the information contained within spectra.

### 4.3. Sampling Modes of Fourier-Transform IR (FTIR)

FTIR works by mathematically Fourier-transforming an interferogram into an actual spectrum. In principle, specific frequencies of IR energy are selectively absorbed by sample constituents, which triggers atomic vibrations—the bending and stretching of the electric dipole moment—within a molecule and eventually results in the vibrational transition from the ground state to an excited vibrational state. These vibrational transitions are associated with corresponding bonding or molecular compositions. They can be interpreted both qualitatively and quantitatively based on the band positions, intensities, shapes, and widths, thereby providing cancer-specific biomarkers with distinctive spectral fingerprints [101]. 

**Figure 2:**
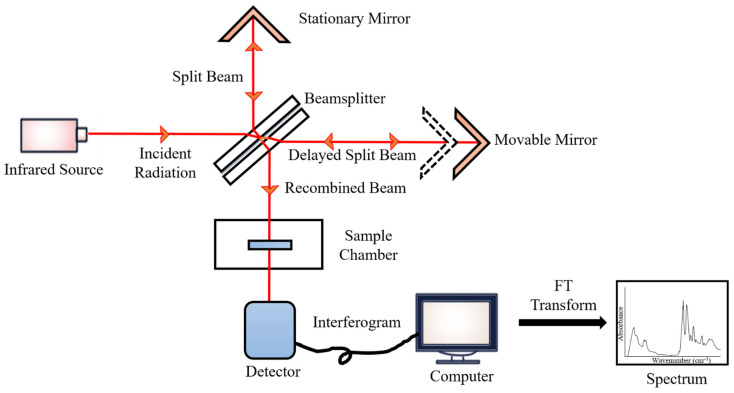
FTIR block diagram.

During the measurements, the emitted IR energy passes through the interferometer along the optical path, where encoding of the spectral signals from the infrared frequencies takes place. The interferometer captures the spectral signals from the infrared frequencies. The interferogram, the resulting signal, is then transmitted through or reflected off the sample surface. The specific energy wavelengths, which represent a sample’s unique molecular characteristics, are absorbed. Eventually, the beam carrying molecular information then passes through the detector, and the measured signals are directed to a processing computer for Fourier transformation of the energy signals (Figure 2) [102].

FTIR spectroscopy has rapidly expanded beyond the essential structural characterization of molecules because of its inherent fundamental principles, simplicity of operation, and analytical sophistication. This technology provides a quick, label-free, and chemically specific examination of non-destructive biological materials with minimal sample preparation and processes, as well as qualitative and quantitative data in the form of reproducible IR spectra. FTIR spectroscopy is a cost-effective and economically sustainable tool for clinical research due to its low analytical cost and minimum reagent utilization during the analysis process. The application of FTIR spectroscopy in biofluid studies makes use of many sampling modes; the main sampling modes of FTIR spectroscopy are transmission, transflection, and attenuated total reflectance (ATR) [103].

In transmission-based sampling mode, IR radiation traverses a sample and a substrate such as calcium fluoride (CaF_2_) [79]. However, as the beam passes through an IR-absorbing sample, this technique requires an optimal pathlength to achieve quality spectra, which is typically specified at 1–20 μm to prevent saturation of the signal and non-Beer–Lambert-like behavior [101]; while in the case of aqueous biological samples, a shorter pathlength is required—not exceeding 6–10 μm—to account for the strong IR-absorbing water molecules [98]. The intensities of the IR bands are nevertheless limited by such short pathlengths. The implementation of transmission FTIR spectroscopy in clinics is rather challenging for aqueous or wet biological samples as their spectral reproducibility can be affected by spacer thickness, surface interactions, the presence of air bubbles in the sample, as well as the hydration level of the sample [101].

Transflection sampling mode functions by transmitting IR radiation through the sample deposited on an IR-reflecting substrate. Some of the incident radiation is reflected specularly from the surface; while most of the radiation is projected to the underlying reflecting substrate and reflected off the substrate through the sample [101]. The transflected radiation is detected and enables the molecular classification of the sample. Notably, the increased pathlength in transflection spectroscopy results in much larger absorption bands in the resultant IR spectra compared to those obtained in transmission and ATR sampling modes [101]. However, transflection FTIR may not be the best option in the study of biological samples. The increased sample pathlength makes it more susceptible to IR absorption by water molecules, in particular for studies involving wet biofluids. In addition, transflection FTIR spectroscopy is more prone to baseline effects due to the significant scattering effects, with resonant Mie scattering being the most prominent effect, resulting in smaller signal-to-noise ratio (SNR). According to Mie theory, the scattering effect occurs when the wavelength of the interrogating radiation is approximately the same size as the scattering particle of tissue or cell samples, which feature a high contrast in refractive index [104]. For instance, the nucleus can give rise to Mie-type scattering and spectral properties with non-Beer–Lambert absorption behavior because of its same size as the wavelength of the IR radiation [105]. Moreover, biological samples, even single cells, might have non-uniformity in size and shape that can give rise to a different extent of the scattering effect.

In addition, many studies have demonstrated that transflection FTIR spectroscopy is prone to the electric field standing wave (EFSW) effect [106,107,108]. An EFSW occurs at reflective metallic-like surfaces when the reflected radiation interferes with the incident radiation. EFSW can cause non-Beer–Lambert-like behavior, which results in non-linear spectral distortion with increasing thickness of the sample. The findings reported by Filik et al. [108] showed that variations in the thickness of bovine serum albumin (BSA) films, ranging from 200 to 1200 nm, gave rise to a different extent of the EFSW artifact, which exerted a significant effect on the transflection spectra by having a non-linear relationship with the IR wavelength. However, the spectral artifacts arising from the effects of light scattering and EFSW can be corrected by applying correction algorithms [109].

The issues addressed in transmission and transflection FTIR spectroscopy could be surpassed by using ATR-FTIR spectroscopy. ATR-FTIR relies on the interaction between an internal reflection element (IRE) comprising an infrared transparent material with a high refractive index—ATR crystals such as germanium, zinc selenide, zinc sulfide, or diamond—and a sample placed on the surface of the IRE [90]. The incident beam is transmitted through the IRE. Total internal reflectance is then instigated, and the IR beam is reflected in the IRE, creating an evanescent wave that protrudes beyond the IRE and penetrates into the surface of the sample for a few micrometers (1–2 μm) to determine its bonding geometry [90]; the wave then loses energy exponentially with distance from the interface of the IRE and the sample, and the resultant radiation is then measured, generating the resulting absorption spectrum [101]. In order to establish total internal reflectance, the critical angle must be minimized by having an IRE with a relatively higher refractive index than that of the sample; the angle of incidence must be greater than the critical angle, or else both the ATR and external refraction will contribute to the resultant spectrum [101]; and the sample must be in direct contact with the ATR crystal [90].

The use of mid-IR spectroscopy for biological studies is challenging because of the presence of water in tissues, cells, or biofluids. Water molecules are capable of absorbing IR radiation over a broad range in the mid-IR region, and can mask the absorption bands corresponding to other biochemical components of the sample. Thus, many studies have been carried out on dried biological samples in order to eliminate the detrimental effect on the resultant spectra caused by water molecules [110,111]. However, spectral differences were observed in fixed or dried samples and the samples in their hydrated or natural aqueous state, which may vary with their hydration state [112,113]. Zohdi et al. [112] demonstrated that the FTIR spectral information obtained from rodent heart and liver tissues preserved through desiccation drying, ethanol substitution, or formalin fixation changed compared to the spectra of fresh hydrated tissue samples, whereby the position and profile of the amide I band varied with the preparation approach and observable intensity loss of lipid absorption occurred when the tissues were hydrated with ethanol.

FTIR coupled with ATR is ideal for biological samples, including biofluids [114,115,116,117], tissues [118,119,120,121], and cells [122,123,124]. One of the main advantages of ATR is based on the use of the surface layer technique, which depends on the interaction between the generated evanescent waves and a few micrometers thick surface layer of the sample. This makes the measurements less likely to be affected by sample thickness, which also allows for simpler sample preparation. However, the sample needs to be at least three- to four-fold thicker than the penetration depth to prevent spectral artefacts with the IRE substrates [90]. Unlike transmission and transflection FTIR spectroscopy, given its small depth of beam penetration due to close proximity between the ATR element and samples, its small effective path length can prevent signal saturation and makes it applicable to aqueous samples, which allows the entire range of the MIR region, including the region corresponding to O-H vibrational modes, to remain accessible [104,125]. ATR-FTIR is thus a more preferable option for studying both dried and hydrated biological samples. Gulley-Stahl et al. [126] demonstrated that ATR-FTIR imaging produced images and resultant spectra with negligible scattering effects even when the biopsied kidney sample contained small amount of mineral that had a high refractive index. ATR-FTIR could thus eliminate spectral artifacts, which have always been an issue in transmission and transflection FTIR. Moreover, in contrast to transmission mode, ATR-FTIR does not need expensive substrates for analysis.

As nano-sized EVs might be well beyond the diffraction limit of IR radiation, a number of studies have exploited the versatility of AFM-IR nano-spectroscopy, which is capable of studying the heterogeneity within EVs at the single vesicle level with nanoscale resolution (<20 nm) and with increased chemical sensitivity to provide spectral information for the characterization of the molecular content of EVs [127,128,129,130]. In general, the sample preparation for AFM-IR spectroscopic analysis includes the deposition of the EV-containing sample onto a solid surface or substrate (e.g., zinc selenide (ZnSe) prism, mica, and gold) to immobilize then sample, followed by drying of the sample for measurements. During the measurements, the laser is aligned with the end of the AFM cantilever, which is in contact with the sample with the AFM tip. The absorbed IR radiation amplifies the thermal expansion signal of the EV sample, and the induced mechanical resonances of the cantilever are then detected by the AFM tip, producing an AFM-IR spectrum from the sample [130].

Despite its ability to analyze nanoscale structure, the sample preparation and instrument setup are rather tedious, time-consuming, and technically challenging. For example, the duration of sample preparation might take hours, and the sample must be securely immobilized to prevent dragging or detachment during the spectroscopic measurements. In addition, the IR laser must be accurately aligned and optimized, and the cantilever must be placed properly prior to acquisition of the AFM-IR spectra in order to obtain reliable spectra from individual EVs. Moreover, substrates such as ZnSe and the laser beam are hazardous, thus special care is required during the measurements [130]. In comparison to AFM-IR, FTIR spectroscopy is easily accessible, cost-effective, faster, and less tedious than AFM-IR, which makes FTIR spectroscopy a more preferable screening tool [131].

## 5. FTIR Spectroscopy in Discovery and Detection of EV-Based Cancer Markers

Cancer development or progression is often associated with changes in biochemical, cellular component, and protein conformational information, hence resulting in modification of the biochemical content in tumor-derived EVs. The alterations in the biochemical compositions could give rise to spectral modifications, such as changes in relative intensity, shape, or relative band position. Taken together, these spectral differences unveil a set of signature spectral biomarkers for cell differentiation.

Some authors have studied the changes in biochemical components of EV samples using a ratiometric approach to evaluate the ratios between the absorbance of selected bands. Direct use of spectral band absorbance values is not preferable due to possible experimental artifacts attributed to factors such as variations in sample thickness, sample purity, etc., during EV isolation [132]. Interestingly, different ratio mixtures have been established by authors to quantify the molecular content of samples. For instance, the lipid/protein ratio was able to differentiate between non-malignant, malignant, and metastatic breast cancer cells [133]; the protein/lipid ratio could differentiate EV subpopulations [89]; the integral (area covered) ratios of amide I/amide II and β–sheet/α–helix discriminated a mouse model of non-Hodgkin lymphoma and melanoma from the healthy mouse model [100]; the spectral intensity ratios of lipid/protein, phosphate/carbohydrate, and RNA/DNA were used to classify ovarian carcinoma subtypes [134]; the ratios of protein/lipid, RNA/lipid, and protein glycogen differentiated cells at different growth phases [135]; etc. Very often, the computed ratios were presented using univariate analyses, such as box plots [32].

### 5.1. Variations of Biochemical Composition in Cancer-Derived EVs

FTIR spectroscopy has been employed to identify EV subpopulations enriched from the same cell. It was first introduced by Osteikoetxea et al., who used the protein-to-lipid ratio to characterize different EV subpopulations [136]. Mihály et al. [137] also performed quantitative analysis using the spectroscopic protein-to-lipid ratio parameter based on relative intensity of amide I and CH stretching bands in IR spectra to differentiate between different EV subpopulations isolated from Jurkat T-cells, namely exosomes, ectosomes, and apoptotic bodies. Their findings revealed that the apoptotic body appeared to have the highest ratio, followed by exosomes, and the lowest ratio was in ectosomes. Similar findings have also been reported wherein a higher spectroscopic protein-to-lipid ratio was shown in melanoma-derived exosomes than in isolated ectosomes [89].

In addition to the protein-to-lipid ratio, different ratio mixtures have also been utilized to differentiate between malignant and non-malignant IR spectra. For example, Di Santo et al. [32] reported variations in the molecular contents of serum EVs enriched from hepatocellular carcinoma (HCC) patients, which were highlighted by spectral variations in the band shape and relative intensity of the protein (amide I and II), nucleic acid, carbohydrate, as well as lipid CH stretching bands. In detail, HCC patient-derived EVs showed an increase in the intensity of the band corresponding to lipid CH stretching vibration, together with a decrease in the intensity of protein, nucleic acid, and carbohydrate bands. These changes were captured by computing the spectral ratio of the molecular components within EVs of the cancer patients and healthy controls, including the lipid-to-protein, lipid-to-carbohydrates, and lipid-to-nucleic acid ratios, which quantitatively measured the relative amount of the biomolecules in the analyzed EV samples. The results showed that the lipid-to-protein and lipid-to-nucleic acid ratios were much higher in HCC patients than in healthy controls. The spectral differences unveiled the potential of using protein, RNA, and lipid contents of EVs as HCC biomarkers [32]. Moreover, Zlotogorski-Hurvits et al. [4] also reported spectral differences of salivary exosomes between oral cancer patients and healthy controls. This study introduced that the relative intensity of the bands between glycogen or carbohydrate and nucleic acids, the asymmetric and symmetric CH stretching vibrations of CH_2_ and CH_3_ methylene groups; and the CH bending vibration bonds in acyl residues of lipids or amines and asymmetric CH stretching vibration of CH_2_ methylene group could be used as the fingerprint FTIR signature for oral cancers [4].

**Figure 3:**
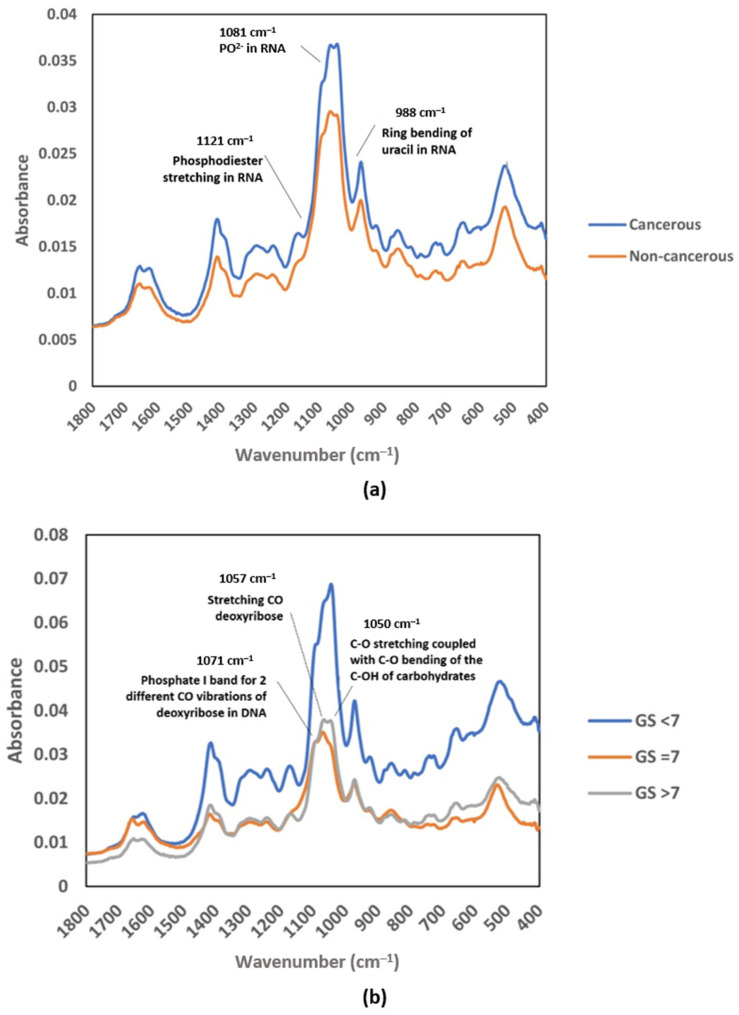
Average raw FTIR spectra of urinary EVs in the MIR fingerprint region: (**a**) cancerous patients and non-cancerous individuals; (**b**) Gleason score groups (GS < 7, GS = 7, and GS > 7). Reproduced with permission from ref. [34], *Membranes*; published by MDPI, 2021.

In a study of prostate cancer (PCa), instead of using the functional group region, Yap et al. introduced the possibility of using the fingerprint region from the average mid-IR spectrum, specifically from 1419 to 968 cm^−1^, to be developed as a complementary test for future screening of cancer. Figure 3 shows that the absorbance spectrum for EVs isolated from urine samples of PCa patients was higher at a few absorption bands than that of healthy subjects, including bands at wavenumbers of approximately 1412, 1052, and 968 cm^−1^. The authors were able to identify the main absorption bands contributing to the cancer classification by performing partial least square (PLS) analysis, including the absorption bands corresponding to vibration in RNA (1121, 1081, and 988 cm^−1^) (Figure 3a). The PLS analysis also revealed that the bands corresponding to CO bond vibration in carbohydrate and DNA (1071, 1057, and 1050 cm^−1^) contributed the most in the discrimination of different stages of prostate cancer (Figure 3b) [34].

Cancer screening and detection at early stages or in preneoplastic states are important to improve curability and the overall patient survival rate. However, in many cases, patients remain asymptomatic and miss the ideal timing for medical treatment. The delay in cancer detection makes the treatment increasingly challenging and complex due to the progression of cancer stages and metastasis [138]. Interestingly, it was highlighted that tumors in different malignancies shared different biochemical and IR spectral profiles in their EV subpopulations. Recently, Stępień et al. [89] revealed the biochemical modifications in melanoma-derived EV subpopulations—exosomes and ectosomes—particularly in their lipid compositions due to subtle changes shown in the IR absorption bands corresponding to the lipid absorption regions between melanoma subtypes. They then established multiple lipid ratio parameters to reveal the lipid composition of the EVs more thoroughly, including the saturated-to-unsaturated fatty acids (Fas) ratio (SFA/UFA), acyl chain length (ACL), and lipid-to-protein ratio for paternal cells and their derivative EVs. The parameters, notably the saturated-to-unsaturated fatty acids ratio, were lowest in the metastatic cutaneous melanoma-derived ectosomes compared to those derived from normal melanocytes and primary melanoma cell lines, suggesting that ectosomes can serve as a potential source of lipid biomarkers for pathology.

In addition, FTIR spectroscopy has the potential to distinguish multidrug-resistant cancer cells from drug-sensitive cells [139]. Spectral variations were reported in the band regions around 1650–1545 cm^−1^ in the spectra profiles of EVs derived from drug-sensitive and -resistant osteosarcoma cells. In detail, the drug-sensitive EV population was defined by the band positioned at 1601 cm^−1^, together with a band shift at 1649 cm^−1^, which might be attributed to modifications in the amide I band and alterations in protein conformation since the amide I band often correlates to different protein secondary structures. The authors also revealed a significant difference between the EVs secreted by both drug-resistant and -sensitive cells by accessing the spectroscopic protein-to-lipid ratio [139].

The potential of FTIR spectroscopy to serve as a screening method for cancer biomarkers was further confirmed by the findings of Paul et al. [131]. In the study, by using FTIR spectroscopy as a complementary spectroscopic tool to AFM-IR spectroscopy, hyaluronan (HA) was identified as a biomarker for colon cancer-derived EVs. Interestingly, when comparing the spectra of pure HA and cancerous EVs, a similar spectral profile was observed, comprising all six signature peaks of HA with similar peak position and relative intensities, including the band regions corresponding to amide or NH wagging (622 and 1113 cm^−1^), C-O and C=O groups in fatty ether (1407 cm^−1^), amide II (1639 cm^−1^), C-H stretching (3005 cm^−1^), and O-H stretching (3137 cm^−1^). Surprisingly, none of the signature peaks of HA were observed in normal colon epithelial cells. The findings proved that FTIR can effectively identify key biomarkers from the EVs.

### 5.2. Structural Changes of Proteins in Cancer-Derived EVs

As reviewed above, hydrogen bonding of different strengths affects the C=O vibration absorption frequency, giving rise to various absorption properties in polypeptides for protein secondary conformation components [99]. Cancer is often associated with modification by mutations and the selection of cells. A mutation in the amino acid sequence may give rise to a structural change in a protein or polypeptide [140]. Evaluation of the conformational changes of EV-derived proteins may provide researchers with a wealth of information in terms of α-helixes, β-sheets, random coils, etc.

Studies have demonstrated the potential of exploiting an EV secondary structural signature as a characteristic biomarker for cancer screening. Findings suggested that malignant cells secreted a higher content of β-sheet-rich protein through EVs than non-malignant cells. For instance, an enhancement of α-helix-rich proteins was observed in healthy sera- and plasma-derived EVs from the deconvoluted amide I band in IR spectra, in contrast to the elevation of β-sheet-rich protein levels in EVs isolated from cancer patients, which may be attributed to systemic alterations in the blood caused by progressive malignant disease [31]. Similar findings were reported by Rasuleva et al. [9]. The second derivative IR spectra of the amide I band were used to explore the secondary conformation of proteins of PDAC tumor-derived EVs. Spectral variations were identified in the IR band components corresponding to α-helix and β-sheet conformations. Quantitative analysis by integrating the corresponding band showed that the ratio of the β-sheet-to-α-helix band was considerably higher in cancer-derived EVs than in normal EVs, suggesting enriched β-sheet proteins in EVs originating from PDAC tumor cells [9].

In addition to alterations in the β-sheet content, Stępień et al. [89] also revealed changes in the percentage of α-helix, β-sheet, inter β-sheet, and random coil content between proteins present in exosomes and ectosomes originating from normal melanoma cells, primary, and metastatic melanocytes using ATR-FTIR spectroscopy. They reported significant variations in the random coil structure content, with the highest random coil content found in ectosomes from metastatic WM266-4 and exosomes from primary WM115 cell lines [89].

In short, these findings prove that information about the distinct pro-tumorigenic content in cancer-derived EVs can be acquired using FTIR spectroscopy, which is reflected in the generated spectra and associated with alterations in relative band intensity, band shape, or band shift. In many cases, the biochemical changes in EVs, including nucleic acids, lipids, and proteins, as well as structural variations in EV-associated proteins, between malignant and non-malignant samples are revealed quantitatively, namely by using different appropriate ratio mixtures. Remarkably, there is no consistent pattern of variations among the samples, suggesting that spectral changes could be exclusive to each cell type, hence showing the potential of developing spectral biomarkers for cancer detection.

## 6. Machine Learning Assisted Analysis of IR Spectral Data

In 1950, Alan Turing first proposed that a computer can learn and become artificially intelligent if it can mimic or simulate human intelligence to perform tasks [141]. Histopathology is the gold standard for cancer diagnosis, but its accuracy is inherently susceptible to operator subjectivity. It is thus important to adopt AI-driven systems in the pathology domain. In 1993, a highly accurate machine learning-based computer-aided detection (CAD) model was established for the diagnosis of breast tumors [142]. Since then, there has been a massive acceleration in the growth of AI models in clinical applications and studies, primarily in clinical decision support [143,144], prediction of the risk of disease [145,146], disease diagnosis [147,148,149], etc. In recent years, many researchers have started to implement AI-assisted models in the spectral analysis of medical diagnosis in order to improve the diagnosis of diseases. 

Given the complexity of the molecular composition on the EV surface and EV derivatives, each generated spectrum can exhibit highly heterogeneous peak compositions, thereby displaying a diverse and complex spectral dataset with overlapping absorption of the main biomolecules. In addition, the spectral variations between different groups, such as malignant and non-malignant samples, are often very subtle. Sample replicates or even the same sample at different time points may exhibit spectral variations due to unavoidable biological changes in the samples, sample degradation, variations in sample preparation, purity [150], etc. In such a complex condition, traditional peak assignment and univariate methods such as box plot analysis are insufficient in assessing the complex spectra of different groups. The complex spectra make traditional band assignment difficult and potentially misleading. At the same time, univariate approaches are restricted to small datasets. They are limited to one variable, such as variation in relative intensity or relative band position (spectral shift) in an IR spectrum, which causes significant information loss [32,151]. To address these issues, computationally aided analysis seems necessary to support the interpretation of multivariate spectral datasets. Chemometrics and machine learning may truly reveal underlying spectral information and permit a more robust classification [151,152].

### 6.1. Chemometrics and Machine Learning

Chemometrics have found widespread use in handling spectroscopic data. Chemometrics can be applied to detect and extract information from subtle changes in IR spectra generated from different samples using mathematical and statistical approaches. Many chemometric methods can be achieved with machine learning approaches, including feature or dimensionality reduction, sample clustering, sample classifying with a classification model, or predicting the samples’ quantitative properties with a regression model [150]. Machine learning is a subset of artificial intelligence (AI), which involves the application of algorithms to learn and improve performance from past experience. Machine learning-based techniques allow the creation of models to review large sizes of multivariate data and produce an automated output that humans can hardly accomplish in an automated manner [153].

Machine learning can be divided into two subcategories: unsupervised and supervised learning. In this context, unsupervised learning, such as principal component analysis (PCA), is used to create a model of the observed patterns. Unsupervised learning deals with unlabeled data and is thus exploratory. The system explores the data by reducing the dimensionality of the spectral datasets and draws inferences from the datasets. It reveals hidden patterns and trends from the unlabeled data, eventually creating clusters based on similar characteristics. On the contrary, supervised learning, such as random forest, decision trees, SVM, etc., aims to create predictive models from a given input pattern. Supervised algorithms highly depend on the information provided by labeled data or pre-determined classifications, such as the histopathological diagnosis. A model is developed on a known training dataset, and its performance is then evaluated in terms of accuracy, specificity, sensitivity, etc. by comparing the output class or output value (for classification and regression, respectively) to the true categories of the validation dataset [55,151,152,154]. If errors are recognized, the model can be modified and corrected accordingly to improve its performance.

### 6.2. Machine Learning of FTIR-Based EV Analysis for Cancer Detection

**Figure 4:**
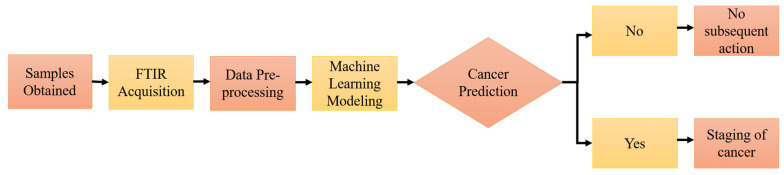
Supervised machine learning workflow. Reproduced with permission from ref. [155], *Diagnostics*; published by MDPI, 2022.

Machine learning models have shown outstanding diagnostic performance in cancer studies, enabling the prediction of the probability or risk that a patient currently has cancer. In order to establish a well-performing machine learning predictive model, pre-processing of the raw FTIR spectral data has become an essential part of chemometric modeling, helping to transform the raw data into a structured and numeric format, hence facilitating the learning process of the applied machine learning algorithms. Nsugbe et al. [155] investigated the application of the linear series decomposition learner (LSDL) approach to minimize the uncertainty and redundancy within the FTIR signal derived from urinary EVs, followed by nine types of machine learning modeling in the prediction of prostate cancer, including decision tree, linear and quadratic variants of discriminant analysis, logistic regression, K-nearest neighbors (KNN), and SVM. The results suggested that the proposed modeling approach could be employed for clinical prostate cancer prediction. Further cancer-stage prediction investigations also showed high accuracy across various metrics studied, demonstrating the capability of urine sample analysis in conjunction with the presented modeling approaches. Figure 4 shows the operational flow of machine learning modeling [155].

Robust and accurate cancer classification is crucial in order for patients to receive correct and effective cancer treatment. A study confirmed that the combination of ATR-FTIR and supervised learning models could permit robust classification of cancerous salivary exosomes. Two discrimination models, principal component analysis-linear discriminant analysis (PCA-LDA) and SVM classification, were developed to classify salivary exosomes derived from oral cancer patients and healthy controls. The discriminant PCA-LDA plot showed successful classification of the two groups with high sensitivity, specificity, and accuracy of 100%, 89%, and 95%, respectively, with only one healthy individual being misdiagnosed as a cancer patient. Remarkably, oral cancers at both the early- and late-stage were recognized in the same cluster, hence revealing the potential application of FTIR as a diagnostic tool for the early detection of oral cancer and monitoring patients with oral precancerous disorders. The SVM discriminant model also showed separation between the cancer patients and healthy controls with training accuracy and cross-validation accuracy of 100% and 89%, respectively [4].

In many cases, different predictive models are trained and evaluated for their performance with the same dataset before deploying the final model to ensure that they are suitable for their given tasks, for instance, the diagnostic prediction of cancer and cancer staging [155]. Uthamacumaran et al. [156] evaluated the classification performance of three different machine learning algorithm models in the discrimination of EVs isolated from the blood sera of different cancer patients (breast, pancreatic, colorectal, and hepatocellular cancers). Both the AdaBoost random forest classifier and decision trees displayed excellent classification performance, with accuracy near or higher than 90%. However, the SVM classification model displayed poor classification performance, with a classification accuracy of 57.14%, five-fold cross-validation score of 75.00 ± 25.00, mean square error of 0.4, and AUC value of 0.50 in the analyzed FTIR spectra. This pointed out that the SVM model might not be best suited for classification purposes in this study.

Remarkably, spectral biomarkers were demonstrated to outperform the classification ability of the conventional HCC circulating biomarkers—alpha-fetoprotein (AFP) and protein induced by the absence of vitamin K or antagonist-II (PIVKA-II)—which confirmed the diagnostic potential of spectral biomarkers in cancer. By using a PCA–LDA discrimination model, several potential spectral biomarkers for HCC diagnosis were identified, including the shape of the bands corresponding to amide I and II, lipid CH stretching, and lipid-ester vibrations. The classification performance of different spectral biomarker subsets, either alone or combined, as well as the two HCC biomarkers, were accessed using stepwise multivariate logistic regression, which was then followed by Receiving Operator Curve (ROC) analysis. Based on the ROC analysis, the combined spectral biomarkers achieved a superior diagnostic performance (AUC = 0.91, sensitivity = 1, and specificity = 0.76) compared to AFP (AUC = 0.81) and PIVKA-II (AUC = 0.86) [32].

## 7. Conclusions and Future Perspectives

EVs have emerged as a valuable source of disease biomarkers for liquid biopsy due to their high abundance and stability in biofluids, as well as the representative molecular profile of their parental tumor cells [7,38]. A wealth of proof-of-concept studies have demonstrated the potential of using FTIR spectroscopy as an exploitable tool for label-free molecular profiling of EVs. FTIR spectroscopy can be used as a rapid, cost-effective, reproducible, and effective diagnostic tool, enabling automated classification through the implementation of multivariate analysis and machine learning with remarkable statistical outcomes (Table 4). This technique is thus well suited to trace and monitor alterations in the cellular state or phenotypes by detecting biochemical changes in EVs, as well as conformational changes in biomolecules, which are less readily accessible using other detection techniques [10]. The research findings are driving the development of diagnostic FTIR assays. They may one day have extensive utility in cancer research to discover novel disease biomarkers and achieve translation into clinics for early detection.

However, the limited numbers of samples used in studies often limit the development of new technologies. The small sample sizes used in FTIR based-EV studies makes it necessary to conduct studies or trials with larger numbers of samples to obtain more robust classification models and ascertain the true clinical diagnostic performance, especially when the statistical outcomes of the machine learning algorithms is highly influenced by the number of samples used in the training and validation of these computational models [86]. Additionally, it is crucial to develop and test multiple classification models suitable for the high-throughput analysis of FTIR spectra of EVs because the models can provide physicians with a direct diagnosis by automatically analyzing the spectral datasets rather than a biophysical parameter that requires further interpretation [32].

Furthermore, the lack of rapid and standardized approaches for EV investigations, particularly in their isolation and characterization procedures, significantly limits the process of translating EV-based biomarkers into the clinic. Conventional EV-isolation methods, such as ultracentrifugation, multistep filtration, size-exclusion chromatography, etc. [10], and easy-to-use commercial isolation kits have been used in EV-related studies [157,158,159]. However, it was reported that the efficiency, purity, and yield of EV isolation; types of isolated subpopulations; and their molecular contents varied with the isolation and purification methods [158,159,160,161,162,163], as well as the type of sample [162] used in the studies. Therefore, standardization of biofluid sample processing procedures (isolation and purification) is needed to accelerate the process of their clinical implementation.

## Figures and Tables

**Table 1:** Infrared Regions.

IR Region	Wavelength (µm)	Wavenumber (cm^−1^)
Near	0.78–2.5	12,500–4000
Mid	2.5–25	4000–400
Far	25–1000	400–10

**Table 2:** Assignments of prominent peaks observed in biological IR spectra.

Wavenumber (cm^−1^)	Vibrational Mode	References
3300, 3298, 3290, 3285	Amide A, which is attributed to peptide N-H stretching vibrations, overlapped with -OH stretching	[89,91,92,93]
3100, 3078	Amide B, which is attributed to peptide N-H stretching vibrations	[89,91]
2959	Asymmetric CH_3_ stretching vibration of acyl chains	[93]
2924, 2921	Asymmetric stretching vibrations of the lipid acyl CH_2_ groups	[4,92,93]
2872	Symmetric CH_3_ stretching vibration of the lipid acyl chains	[93]
2851, 2850	Symmetric stretching vibrations of the lipid acyl CH_2_ groups	[4,92,93]
1745, 1743, 1740, 1738	Saturated ester C=O stretch of lipids, phospholipids, triglycerides, and cholesterol esters	[4,92,93,94]
1657, 1650, 1646	Amide I, which arises mainly from C=O stretching vibrations of the protein peptide backbone, coupled weakly with C-N stretch, N-H bend, and C-N-C deformation	[89,91,92,93,94]
1550, 1546, 1540, 1537	Amide II, which originates from N-H vibrations of the peptide groups with C-N stretching	[89,91,92,93,94]
1448	Bending (scissoring) vibration of lipid acyl CH_2_ groups	[93]
1402	Symmetric stretching vibrations of COO- in fatty acids and amino acids	[93]
1314, 1300	Amide III, which is attributed to C-N stretching and N-H in-plane bending, often with deformation vibrations of C-H and N-H	[91,93]
1236	PO_2_^−^ antisymmetric stretch of phospholipids and nucleic acids	[93]
1156	CO-O-C antisymmetric stretching of glycogen and nucleic acids; and C-O stretching from alcohol groups of glycogen and lipids	[93]
1080, 1072	PO_2_^−^ symmetric stretch of phospholipids and nucleic acids	[4,93]
1033	–CH_2_OH groups and the C-O stretching vibration coupled with C-O bending of the C-OH groups of carbohydrates	[4]

**Table 3:** Assignment of protein secondary structure based on the IR amide I band analysis.

Wavenumber (cm^−1^)	Band Assignment
1610	Sidechain
1630	β-sheet
1645, 1648	Random coil
1652	α-helix
1682	β-turn
1690	β anti-parallel sheet

**Table 4:** A summary of the cancer-derived EVs studies using FTIR spectroscopy discussed in this review.

Study	Cancer Type	EV Type	Wavenumber (cm^−1^)	Band Assignment/Vibrational Mode	Analysis Methods
[4]	Oral Cancer	Salivary exosomes	2924, 2854	Asymmetric and symmetric C-H stretching vibrations of lipid CH_2_ and CH_3_ methylene groups	Ratiometric, PCA–LDA, and SVM
			1743	C=O stretching vibration in lipids	
			1547, 1543	Amide II	
			1404	CH bending vibration bonds in acyl residues of lipids/amines	
			1072	Symmetric stretching of nucleic acid phosphodiester groups	
			1033	Vibrational mode of –CH_2_OH groups, C-O stretching vibration and C-O bending of carbohydrates	
[9]	Pancreatic cancer	PDAC cell- and sera-derived EVs	1700–1600	Amide I	Ratiometric
			1653 ^1^	α-helix ^2^	
			1644 ^1^, 1635 ^1^	β-sheet ^2^	
[31]	Prostate cancer	Sera- and plasma-derived exosomes and ectosomes	3298	Amide A	Observation
			1656	Amide I	
			1544	Amide II	
			1667–1686 ^1^	β-turns ^2^	
			1620–1640 ^1^, 1670–1695 ^1^	β-sheets ^2^	
			1648–1657 ^1^	α-helices ^2^ and random coils ^2^	
[32]	Hepatocellular cancer	Sera-derived EVs	1200–1000	Carbohydrate and nucleic acid band	Ratiometric, PCA–LDA, and multivariate logistic regression
			3000–2800	Lipid C-H stretching vibration	
			1735	C=O stretching of the purine base and lipid-related ester group	
[34]	Prostate cancer	Urinary EVs	988, 1121, 1081	Phosphodiester stretching from nucleic acids	PCA and PLS
			1050, 1057, 1071	CO bond vibration in carbohydrate and DNA	
[89]	Melanoma	Melanoma cell- derived exosomes and ectosomes	3290	Amide A	Ratiometric
			3078	Amide B	
			3006	Unsaturated fatty acid	
			3000–2800, 1396	Lipid acyl chain	
			2922, 2853	Saturated fatty acid	
			1743, 1728	Lipid interfacial region	
			1650	Amide I	
			1540	Amide II	
			1300–1000	Lipid head group	
			970	DNA	
[131]	Colorectal cancer	Cell-derived EVs	3137	OH stretching of carboxylic acid group	Observation
			3124, 3005	C-H stretching of alkane group	
			1639	Amide II	
			1407	Symmetric and asymmetric vibration of COO−	
			1244, 1113	C-O group with C=O in fatty ether	
			622	Amide/NH wagging	
[137]	Leukemia	Leukemia cell-derived EVs	3285	Amide A	Ratiometric
			2924, 2850	Antisymmetric and symmetric stretching vibrations of lipid acyl CH_2_ groups	
			1740–1725	C=O stretching of lipid-related ester bonds	
			1650	Amide I	
			1540	Amide II	
			1453	Bending (scissoring) vibration of lipid acyl CH_2_ groups	
			1394	Bending vibrations of CH_3_ groups of lipid and protein	
			1676 ^1^	β-turn ^2^	
			1660 ^1^	Triple-helix structure ^2^	
			1640 ^1^	Random coils ^2^	
			1635 ^1^	β-sheets ^2^	
			1627 ^1^	Non-native intermolecular β-sheets ^2^	
[139]	Osteosarcoma	Cell-derived EVs	2930, 2852	Acyl group vibrations	Ratiometric
			1780–1550	In-plane vibrations of double bonds of nucleic acid bases	
			1738	Ester groups of phospholipids, triglycerides, and cholesterol esters	
			1649	Amide I	
			1550–1270	Deformation vibrations of nucleic acid bases and sugar vibrations	
			1455	Amide II	
			1407	Symmetric and asymmetric vibration of COO−	
			1270–1000	Vibrations of –PO_2_−	
			1240, 1080	Symmetric and asymmetric stretching of the nucleic acid phosphodiester groups	
			1040	Polysaccharides	
			1000–780	Vibrations of sugar-phosphate backbone	
			992–986	Ribose phosphate main chain	
			966	Stretching vibration of the DNA backbone	
[156]	Pancreatic, melanoma, colorectal, and breast cancers	Sera-derived EVs	3400–3200	OH stretch	PCA, AdaBoost random forest classifier, decision trees, and SVM
			3010–2850	C-H stretching vibration	
			3070	-	
			1120	-	

^1^ Wavenumber originating from deconvoluted amide I absorption band. ^2^ Protein secondary structure.

## Data Availability

Not applicable.
